# Navigating Long‐Term Co‐Creative Research With Young Adults Diagnosed With Cancer: A Qualitative Study

**DOI:** 10.1111/hex.70713

**Published:** 2026-06-03

**Authors:** Sarah Marklund, Rebecca Skog, Lars Sjödin, Johanna Rose, Marit Silén, Lena Wettergren, Claudia Lampic

**Affiliations:** ^1^ Department of Psychology Umeå University Umeå Sweden; ^2^ Department of Public Health and Caring Sciences Uppsala University Uppsala Sweden; ^3^ Department of Women's and Children's Health Karolinska Institutet Stockholm Sweden

**Keywords:** cancer survivorship, internet‐based intervention, patient engagement, patient involvement, psychosocial intervention

## Abstract

**Introduction:**

Patient and public involvement in cancer research is increasingly recognised as essential for developing interventions that are relevant and meaningful to those affected. However, few studies provide detailed accounts of such collaborations or evaluate its impact. This study aimed to evaluate a long‐term, co‐creative process to refine and improve an internet‐delivered intervention focusing on fertility and sexuality following cancer (Fex‐Can 2.0).

**Methods:**

This qualitative study evaluates the collaboration between patient research partners and researchers involved in a shared working group. A total of 10 young adults (26–39 years), were recruited for via e.g., social media and patient organisations. Seven shared working group meetings were conducted, during which the content of the Fex‐Can 2.0 intervention, and components of the planned evaluation, were revised. Data, including information from impact logs, field notes, and individual interviews with patient research partners (*n* = 4), were analysed using qualitative content analysis.

**Results:**

Three main categories were constructed. The category *Collaborative working process* captured forms of collaboration in the shared working group, highlighting the importance of transparency and feedback for effective collaboration. The category *Group atmosphere* described efforts to create a permissive environment, ultimately fostering emotional safety and engagement. Finally, *Concrete impact* reflects tangible changes to the intervention and future pilot trial, including revisions to language and content, structure of texts, intervention design elements, recruitment and interview guides.

**Conclusions:**

Through structured meeting formats and an iterative process with continuous feedback, the collaboration fostered trust and inclusivity, with patient research partners feeling cared for and able to contribute meaningfully. Their multifaceted input led to concrete improvements to the Fex‐Can 2.0. This study contributes to a growing body of knowledge supporting patient and public involvement in intervention development. Future research should continue to explore long‐term involvement, strategies for recruiting underrepresented groups, and transparently report impact on intervention design and outcomes.

**Patient or Public Contribution:**

A group of young adult patient research partners collaborated with researchers to refine and develop version 2.0 of the Fex‐Can intervention. Contributions from patient research partners included co‐design of the intervention and involvement in reviewing and discussing results from the qualitative content analysis of the present paper.

**Trial Registration:**

Trial Registration: ISRCTN18040643. Registered 12th of December 2024.

**Abbreviations**
PPI: patient and public involvement
PRP: patient research partners
RCT: randomised controlled trial

## 1 Introduction

Patient and public involvement (PPI) is defined as research conducted *by* or *with* patients and/or the public, rather than *for*, *to*, or *about* them [1]. PPI in cancer research has received increased attention over the past decades [2, 3], reflecting a broader trend towards integrating patients' perspectives into healthcare research and practice. This shift is crucial as it acknowledges patients as experts in their own experiences, enhancing the relevance and applicability of the research [1].

Patients and the public can be involved in all phases of research and at various levels of involvement, as described in guidelines and previous research [1, 2, 4, 5]. Several studies have described the involvement of individuals with lived experience in the development of interventions targeting cancer patients [6, 7, 8, 9, 10]. However, only a few have explicitly sought to evaluate the involvement of patients and/or the public in the development process. Nissen et al. [7] evaluated their patient involvement in the development of a psychosocial cancer rehabilitation intervention. The collaboration involved researchers and five patient representatives working together in a shared working group engaged in developing intervention materials, interview guides, and project procedures, including recruitment and participants information. The evaluation demonstrated that patient involvement influenced multiple aspects of the project, including intervention content (e.g., workings of text, visuals, video content), project procedures (e.g., changes in interview guides and recruitment), and researchers' perspectives and ways of thinking about the research project [7]. Across the broader field of health services research, PPI has generally been reported to have positive impact on language, content, design and delivery of interventions [11, 12, 13, 14, 15]. Additionally, results have highlighted that involvement of patient research partners (PRPs) improves enrolment and retention rates in clinical trials [16], as well as acceptability of research [17].

It has been argued that there is a need for more studies describing the process of involving PRPs in research in order to make this type of research better aligned and easier to reproduce and fine‐tune [18, 19, 20]. Further, previous research has highlighted that there is a lack of studies reporting impact of PPI [21, 22], which limits our understanding of how involvement has made a difference [23]. Provision of detailed descriptions of involvement and its impact are essential for advancing the knowledge that can guide researchers in understanding what works and under what circumstances it works [23, 24, 25]. Building on this need, transparent reporting of the context and processes of involvement has been highlighted as crucial for strengthening the PPI evidence base, in line with recommendations from key reporting guidelines [25].

### 1.1 The Fex‐Can Project

The present study was conducted as part of the Fertility and Sexuality following Cancer (Fex‐Can) project that investigates fertility‐related distress and sexual dysfunction among individuals diagnosed with cancer during young adulthood [26] and aims to develop and evaluate internet‐delivered interventions to alleviate such problems [27].

**Figure 1:**
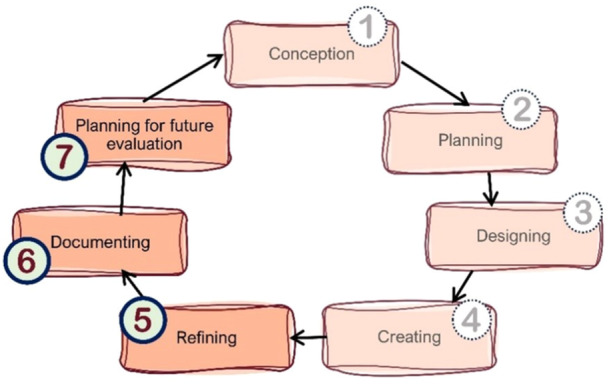
Overview of the research steps when developing an intervention, based on the domains described by O'Cathain et al. [5].

PPI is a core feature of the Fex‐Can project. In 2014, a first collaboration with a group of PRPs was initiated, which continued for over 5 years [28]. During this first collaboration, two internet‐delivered psychoeducational programs were developed, targeting fertility‐related distress (Fex‐Can Fertility) and sexual problems (Fex‐Can Sex), respectively [27, 28, 29]. These interventions were evaluated in two randomised controlled trials (RCTs), with the primary outcomes fertility‐related distress and sexual dysfunction, as well as several secondary psychosocial outcomes (e.g., body image distress, emotional distress, health‐related quality of life) [27]. Although the evaluation demonstrated limited effects on primary and secondary outcomes, participants reported that they felt helped by the intervention [30, 31]. Subsequently, we set out to refine and further develop the intervention and study procedures. To do this, we recruited a new group of PRPs to collaborate with in the development of the next generation of the intervention, Fex‐Can 2.0. The aim was to establish a long‐term collaboration that would continue throughout the relevant steps of the research cycle (i.e., refining, documenting and planning for future evaluation; see Figure 1). The Fex‐Can 2.0 intervention will subsequently be evaluated in an internal pilot trial and full‐scale RCT [32].

### 1.2 Aim

This study aimed to evaluate a long‐term, co‐creative PPI process with regard to engagement and impact on an internet‐delivered psychoeducational intervention. This co‐creative collaboration sought to refine and improve the intervention to alleviate fertility‐related distress and sexual problems following cancer in young adulthood.

## 2 Materials and Methods

### 2.1 Design

This qualitative study presents the evaluation of the collaboration between PRPs and the research team involved in a shared working group. A qualitative design, informed by a constructivist approach, was chosen to enable an in‐depth, context‐dependent understanding of the collaborative processes and how PRPs involvement shaped the development of the intervention. The collaboration included a series of shared working group meetings conducted over an 18‐month period, with intermediate homework assignments (PRPs) and planning/follow‐up sessions (research team). The study uses multimodal data including impact log information from shared working group meetings, field notes from group meetings and research team sessions, and semi‐structured interviews with PRPs. Reporting abides by the GRIPP2‐SF (Guidance for Reporting Involvement of Patients and the Public‐Short Form) checklist [25] and SRQR (Standards for Reporting Qualitative Research) checklist [33]. Ethical approval for the study was granted by the Swedish Ethical Review Authority (record no: 2024‐01576‐02, 2025‐02691‐02).

### 2.2 Predetermined Components

The refinement of the Fex‐Can intervention concerns the final three steps [5, 6, 7] of the intervention development domains described by O'Cathain et al. [5] (Figure 1). Steps 1–4 were completed during the development on the original Fex‐Can programs (Fex‐Can Sex and Fex‐Can Fertility), setting the basis for the Fex‐Can 2.0. This means that the main components of the intervention were determined prior to initiating the present collaboration with PRPs. For example, the intervention was planned to consist of distinct modules including written text and illustrations, exercises, quizzes and reflective questions, and short videos of young adults talking about their experiences, and to include a moderated discussion forum. Further, consultative telephone conversations with each participant at the start and completion of the program were also planned.

### 2.3 The Shared Working Group

The shared working group consisted of 10 PRPs and 4 members of the Fex‐Can research team (S.M., R.S., L.S., and J.R.).

#### 2.3.1 PRPs

**Figure 2:**
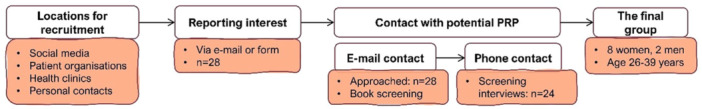
Overview of the recruitment process of the PRP group.

PRPs were recruited through various channels, for example, patient organisations and social media (Figure 2). The aim was to recruit young adults (18–39 years) with a cancer experience, either as survivors or partners of survivors. All recruits were currently experiencing fertility distress and/or sexual problems. The recruits also had to be able to communicate in Swedish and be willing to work on‐site within a group (for at least a year), as well as revise materials at home between meetings.

Interested individuals signed up by contacting the research team via email or by completing a form on Uppsala university's website. Initial contact was then made via email to provide additional information and to set up a screening interview with S.M. or R.S.

Of the 28 individuals who reported interest, 24 individuals responded and agreed to a screening interview. We excluded one individual with experience of being a relative to someone with cancer, but not as a partner. The remainder had contacted us due to themselves having had cancer. The screening interview was conducted in order to offer a verbal description of the project and to allow candidates to ask questions. During the screening, potential PRPs were asked to speak about their situation and their concerns regarding sexuality and/or fertility following cancer. This information was used in an effort to involve people with diverse backgrounds and experiences, including problems/worries, cancer diagnoses, gender, sexual orientation, age, family constellation, and place of residence. A PRP group size of 10 was deemed sufficient to account for potential absence or drop out at meetings while still allowing for an open discussion and relaxed atmosphere with everyone present.

The final PRP group initially consisted of eight women and two men, aged between 26 and 39 years (median: 33.5 years), and diagnosed with (breast [*n* = 5], cervical [*n* = 3], sarcoma [*n* = 1], and oral cavity [*n* = 1]) cancer up to 10 years ago (median: 2 years). A majority of PRPs had a partner, and about half had children. Overall, PRPs represented various backgrounds and life situations, in terms of relationship status, sexual orientation, and family constellations. No partners of survivors were involved in the collaboration.

Over time, two PRPs (women, breast cancer) left the shared working group due to time constraints and travel logistics, and life changes that hindered continued engagement. All PRPs were paid an hourly rate of 200 SEK (appr. 21 USD) for their involvement, and it was made clear that they did not have to participate at each meeting but rather participate to the extent they were willing and available.

#### 2.3.2 Researchers and Reflexivity

Fex‐Can is a multidisciplinary research project group, with backgrounds in medicine, psychology, nursing, physiotherapy, sociology, social work, gender studies, public health and communication. All Fex‐Can research project group members were involved in defining the collaboration's goals, recruitment strategy, and structure. Four of the research project group members have been directly involved during the shared working group meetings—three researchers (a behavioural scientist, a physiotherapist and sociologist, a social worker and licensed healthcare counselor) and a project coordinator, aged 29–39 (S.M., R.S., L.S., and J.R.) (hereafter referred to as research team members). These have been responsible for the planning, implementation, note taking and communication with PRPs throughout. They also brought diverse personal experiences, including various experiences of, for example, gender, chronic illness, parenthood, partnership, and previous co‐creation work. Two of them had also worked clinically with patients, where one had experience in delivering counselling sessions in oncology. At most meetings, three team members attended—two led the workshops, while one took notes and handled logistics.

The research team met regularly to reflect on past meetings with the shared working group, and plan for upcoming ones. One member (R.S.) led the coordination of the collaboration, handling strategic and logistic planning with support from the larger Fex‐Can project group. Prior to each shared working group meeting, R.S. summoned the research team members to set the agenda, plan the session structure and activities, and prepare for the home assignment. These preparatory meetings also provided opportunity to discuss any potential challenges and decisions regarding facilitation strategies for the upcoming session (e.g., group work, speaking rounds, breaks). In addition, reminders were sent out to PRPs about 3 weeks in advance to confirm attendance and arrange travel, followed by their home assignment about 10 days before the meeting. The project coordinator (J.R.) managed travel, lodging, catering, and related logistics.

Two external research personnel were recruited for this study, the interviewer and initial analysist for the interviews with PRPs (M.S., a woman of 46 years, experienced interviewer) and a person (man, 27 years, journalism student) quality checking the transcriptions. These two had no prior relationships with the interview participants and only M.S. would meet with them either via link or in person.

### 2.4 Data Generation

Data generation was based on three sources: impact log information from the shared working group meetings, field notes (by research team members during and after shared working group meetings), and interviews with PRPs.

The impact log template used was inspired by the NIHR Collaboration West Research (ARC West) template [34] and modified by R.S. to fit our needs (Supporting File S1). It was used for taking notes and to reflect on activities and effects of discussions held during the meetings. Apart from practical information (e.g., date, location, participants and absentees), the impact log template covered outcomes from the discussions, including ideas and suggestions brought up during the meetings, the impact of and reflections about such suggestions, and next steps. During meetings either R.S. or J.R. were responsible for filling out the impact log.

Two types of field notes were gathered for this study. First, during each shared working group meeting, a member of the research team documented detailed observations of the meeting context, interactions among participants, and the progression of discussions. In most cases, the research team member who completed the impact log also collected these observational field notes. Second, at the end of each meeting, the research team held a reflective discussion focused on how the meeting had unfolded and what lessons could be drawn for future sessions from the research team's perspective. Field notes from these reflective discussions were documented by S.M.

Individual interviews with PRPs were performed after the 7th shared working group meeting, by the external researcher (M.S.). All eight PRPs were approached and three women and one man (26–39 years old) accepted being interviewed. The interview guide covered five areas: (i) motivation, (ii) recruitment process, (iii) roles and communication, (iv) affect and organisation, and (v) emotional aspects and expectations. The interviews were held via video chat (the preferred space of the interviewee) and lasted 14−24 min (mean interview time: 17.5 min). They were documented using audio recordings and transcribed verbatim. The audio recordings and full transcripts were accessible only to the two external resource persons.

### 2.5 Data Analysis

All data sources were analysed using qualitative content analysis, as described by Graneheim and Lundman [35]. This was chosen as the study is closely connected to social interaction and as qualitative content analysis is especially useful for written data material (e.g., impact log information, field notes), but equally useful for interview data.

All data went through the same steps starting with (i) going through and getting to know the material, (ii) dividing the material into meaning units, (iii) coding the units and (iv) grouping the codes into categories [35]. Impact log information and field notes were analysed by S.M., followed by discussions with R.S. For analysis of the interviews, M.S. made a code tree with preliminary categories, codes and representative raw data, which then was revisited and revised by S.M. who triangulated the findings (still blinded to uncensored raw data). Results from the analyses of the different data sources were then integrated into a larger code tree. Final categories were based on the material of all three data sources. PRPs were asked to review the results in order to ensure that no misinterpretations had occurred and that results corresponded with their experiences of the collaboration.

## 3 Results

**Figure 3:**
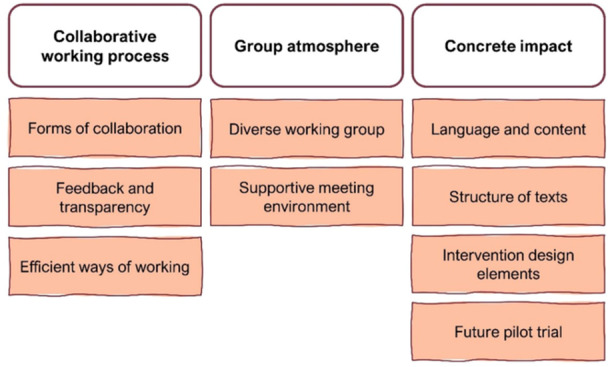
Overview of main categories and subcategories.

The analysis of impact log information, field notes and interviews resulted in the construction of three main categories and nine subcategories (Figure 3). The main category *Collaborative working process* describes how the researchers and PRPs collaborated in the shared working group and includes three subcategories: *Forms for collaboration, Feedback and transparency*, and *Efficient ways of working*. The main category *Group atmosphere* focuses on efforts to create an inclusive and permissive work environment, and includes two subcategories: *Diverse working group* and *Supportive meeting environment*. Finally, the main category *Concrete impact* consists of four subcategories that describe how the collaborative work process resulted in changes to the intervention and its planned evaluation: *Language and Content, Structure of texts, Intervention design elements*, and *Future pilot trial*.

### 3.1 Collaborative Working Process

#### 3.1.1 Forms of Collaboration

The collaboration included preparatory work (research team), home assignments (PRPs) and in‐person meetings at university premises (shared working group). Each meeting had a main theme, for example, introduction to PPI‐research, content and language of the intervention, interactive components, and the future pilot trial. A brief overview of the shared working group meetings is presented in Table 1.

**Table 1:** Themes and content of shared working group meetings.

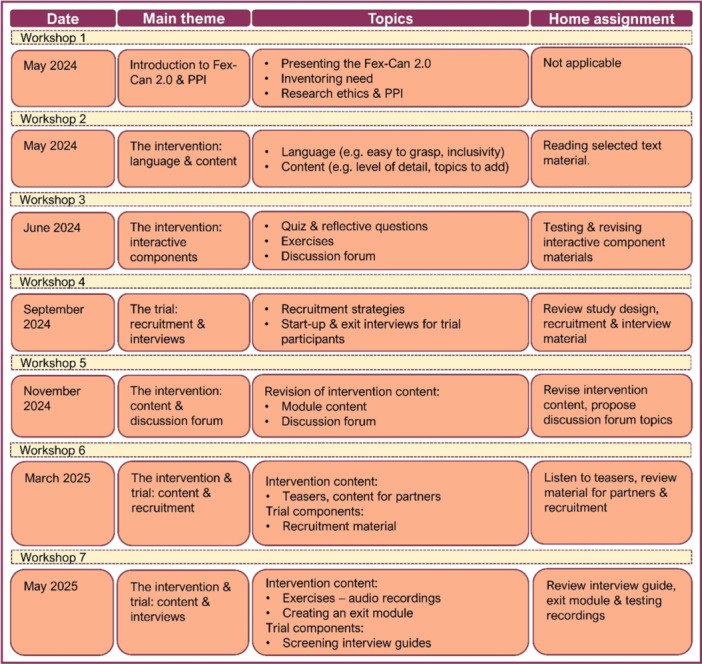

Early meetings of the collaboration were held close together to establish the collaboration, while later meetings were scheduled when specific tasks required collective input. This flexible needs‐based approach supported efficiency. Expectations regarding meeting frequency and variability were discussed throughout the collaboration, and meeting dates were set in consultation with PRPs, emphasising flexible and voluntary participation. Continuity between meetings was maintained through regular e‐mail communication. Each meeting followed a consistent form centred around a main theme and typically two to three key topics. Meetings took place on Saturdays, lasting 4.5–6.5 h, including coffee breaks and a shared lunch. Water, snacks, and warm beverages were always readily available. Meetings were structured as follows: (1) welcome and a brief overview of the day, (2) sharing updates of importance since last meeting, (3) feedback from the research team, (4) lunch, (5) workshop sessions, (6) coffee break and time to recharge, (7) group reflection. Throughout the day PRPs were actively encouraged to change physical positions, eat, drink, and take breaks as needed (e.g., due to pain, tiredness, etc.). Workshops held during the meetings included both full group and smaller group formats, based on home assignments. These assignments varied by meeting but typically included intervention text materials to review and revise, prototype audio files to listen to, exercises to try or reflect on, and descriptions of various study components (e.g., interview guides) to help PRPs prepare for upcoming discussions. The work used an iterative process with continuous reflection, focusing on balancing content development and implementation.

#### 3.1.2 Feedback and Transparency

The meeting structure was designed to collect thoughts and ideas and come up with potential courses of action. After each meeting, the research team met to discuss lessons learned and to identify next steps. The meeting output and reflections were then discussed with the larger Fex‐Can research project group to plan for potential implementation of suggestions raised during shared working group meetings. Based on these discussions and on revisions made to intervention materials, new questions and tasks were formulated for the upcoming meeting and sent to the PRPs in advance. In this way, each meeting built on the previous one, allowing for elevated and in‐depth discussions over time.

To increase agency and feeling of ownership, as well as streamlining efforts within the shared working group, several strategies were put in place to provide PRPs with transparency and context. Firstly, through continuously informing PRPs about what *could* and *could not* be altered, meetings could be constructively focused on what could be changed. For example, suggestions for various functions to be included in the discussion forum were brought forward. However, not all suggestions were possible to implement due to constraints of the internet platform, and this was then openly discussed with the PRPs. Secondly, with suggestions brought forward during meetings that led to adjustments to the intervention, new insights came about in the Fex‐Can project group. In several cases, this resulted in additional adjustments to related content where the same suggestions could be applied. Adjustments made between meetings were reported back to the PRPs at the next meeting, followed by opportunity for questions and comments. This approach was touched upon in the interviews, where PRPs reflected on the discussions held during meetings.

They've started pretty much every meeting with, okay, “the last or the two second last times we have focused on this” and they have summarised and confirmed with us “did we understand you correctly?”. [I've] strongly appreciated that they confirmed with us.

(IP4)

#### 3.1.3 Efficient Ways of Working

The researchers took seriously the important role and responsibilities of providing the PRPs with transparency and consistent updates, something that has been continuously discussed between meetings as noted in field notes. Partly, this was achieved by preparatory home assignments to work on in‐between the shared working group meetings, which increased the insight into and knowledge about upcoming meetings. This further reduced the time spent introducing a topic at the meeting and allowed for more time for discussions.

Since time is limited when we meet it is important that we have prepared too. Listen to some link, read some materials, provide feedback. It you haven't been able to attend you could still provide some feedback on the material. So, I thought that worked very well with how it was organised.

(IP4)

The home assignments, alongside transparency about what could and could not be changed and providing clear rational for decisions, allowed for greater focus of energy and time on the things that the shared working group could impact. This set the frames for our work and allowed PRPs to provide input on things that could lead to real changes.

It has been very clear […] So, we knew what they were looking for. And they were always fast to respond if we had any questions. So, there were never really any unclarities. Just straight on and clear all the time.

(IP2)

### 3.2 Group Atmosphere

#### 3.2.1 Diverse Working Group

Screening interviews conducted during the recruitment of PRPs were key in putting together the group. From field notes and in impact log information, researchers described the screening interviews as a way of getting a good sense of prospective PRPs, and as an opportunity to clearly communicate what the collaboration entailed. In interviews, PRPs further described the screening interview as a positive experience, and expressed appreciation for the conversation:

She [the researcher] described really that it was our experiences, opinions, and thoughts, that were really what they were looking for and that it is really important […] it really made me feel that we are important in this. And I liked that they wanted to have people with their own experiences.

(IP2)

PRPs further reported that the shared working group felt diversified, with people from various circumstances and previous experience. A PRP recalled: ‘And that it's been different people, different backgrounds, different family situations, life situations. And I think it is important to get as many aspects as possible’. (IP4)

Creating diversity within the PRP group was one of the core aims of the screening interviews, which appeared to have at least partly had the intended effect. Still, only two of the PRPs were men, only one reported a non‐heterosexual sexual orientation, all had a higher education, and place of residence was limited to central Sweden (an area including two of the country's largest cities).

#### 3.2.2 Supportive Meeting Environment

The research team aimed to create an environment where ideas would be openly shared and discussed. In working to ensure this climate, discussions were designed to accommodate various preferences regarding co‐creative work, such as working in pairs, small groups, big groups, and individually. The shared working group was also tasked with forming a ‘meeting contract’ at the first meeting, where rules on communication and social environment were suggested, discussed and decided upon by group consensus. For example, the group decided that structured turn‐taking, where each group member get to share their perspective in turn, were a good way to facilitate discussions, to ensure that everyone gets to speak their minds. As described in field notes, researchers decided to actively help one another to remember and uphold these turn‐taking rounds to ensure space for everyone to be heard during meetings, as this is something that is easily forgotten during heated discussions. When differing preferences or needs emerged within the group, researchers aimed to support open dialogue to ensure that all perspectives were heard, and the group then worked collaboratively to identify ideas that balanced these views. In addition, the reflective meetings held among researchers following each shared working group meeting allowed for discussion of any challenges that had emerged and for adjustments to facilitation strategies at subsequent meetings.

Other suggested and accepted rules entailed allowing for strong emotions, accepting all perspectives and experiences as valid and important, and agreeing to avoid discussions on politics to the extent that it was possible. This brought a sense of safety and courage to the PRPs, where it was okay to speak one's mind, discuss, and open up about sensitive subjects. PRPs expressed that: ‘You've felt very safe, to be able to express different opinions and be vulnerable with the heavy things that we are talking about’. (IP1)

Additionally, each shared working group meeting started with a review of the meeting agenda, followed by an opportunity for group members to share how they were doing and whether anything significant had occurred since the previous meeting. Such updates entailed everything from birthdays to newly unearthed cancer diagnoses. To celebrate and commiserate together was an important step to build trust, openness and accountability towards each other. It also built a social connection where respect and compassion were shared. A PRP expressed that ‘[the researchers] have really made us feel welcome and important […] and cared about everyone. And everyone cared about everyone. And it has been a really good dynamic’. (IP1)

Finally, the research team continuously aspired to make sure the group's needs were met, including needs like access to food, restrooms and breaks. Over time, additional needs were brought forward. For example, PRPs expressed a need for new information and tasks to be sent out beforehand and not introduced at the meeting to avoid the stress of having to perform impromptu. This was subsequently implemented at the upcoming meetings. Ideas and suggestions pertaining to the setting or structure of the meetings themselves were further brought up. For example, PRPs requested to shorten the lunch break, in order to increase time available for discussions, which was adapted in the upcoming meeting. However, all suggestions were not possible to implement. Some PRPs suggested that the 1‐day meetings should be lengthened to 2‐day meetings. However, after openly discussing the varying need and want of the group (PRPs and research team), consensus within the group was reached not to move forward with this suggestion.

### 3.3 Concrete Impact

The collaboration resulted in wide‐ranging feedback and input on the intervention language, content, structure, design and future evaluation of Fex‐Can 2.0. Examples of input and subsequent changes applied are presented in Table 2.

**Table 2:** Examples of PRPs impact on theFex‐can 2.0 intervention.

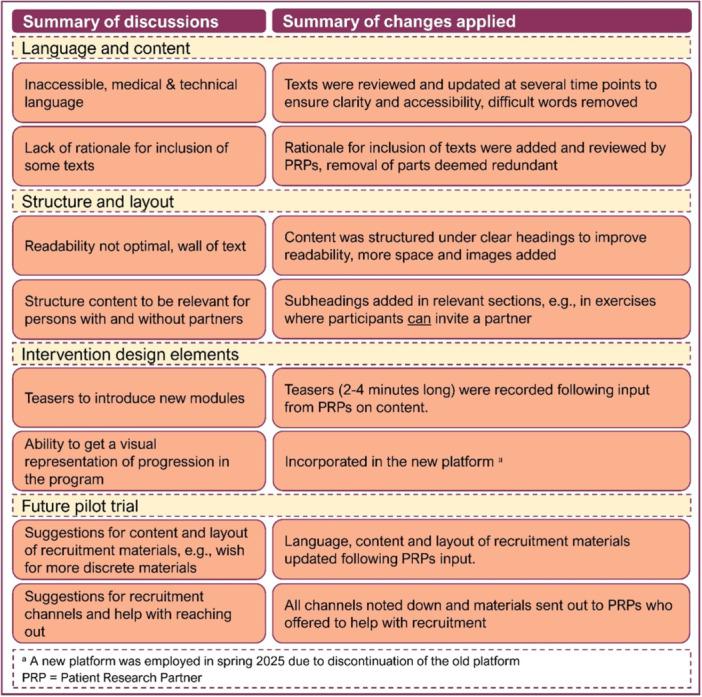

Suggestions and ideas, that were doable and relevant, were translated into actions. For example, the research team rewrote and restructured segments of the content to increase readability by, for example, removing difficult words and incorporating expandable text sections. To make sections more relevant, pedagogical, and tailored towards the needs and wants of the target group, the PRPs read, watched and listened to materials, and tried various exercises. Audio files were added to the material for enhanced attractiveness and inclusion following suggestions of PRPs, and interactive components were reworked. As this work developed, various technical parts were discussed with platform developers for future applications, and for further knowledge of the constrains and opportunities of the platform.

Further, PRPs were also invited to take on an active role within the intervention by moderating the discussion forum included in Fex‐Can 2.0. Additionally, they were invited to contribute to the program through recording videos or audio files sharing their experiences of fertility‐related distress and sexual problems following cancer. A total of five PRPs accepted these roles, which will provide future participants with authentic peer perspectives.

#### 3.3.1 Language and Content

PRPs raised issues with details such as choice of words and visual attractiveness of the intervention, including words or images that risked inadvertently placing blame or upholding norm. An example documented in the impact log, was a suggestion to change from a formulation that asked ‘what the person is doing’ to know more or have less anxiety, to asking ‘what the person would need or want’ to understand or be able to handle their situation better. Another example was mentioned during the interviews, reflecting the impact that PRPs had on the language.

When it's been a clinical or difficult language, we've brought other suggestions on how to say or express this to make it feel more available and relevant. Some words you hardly understood […] in several places, for example, it said “reproduction apparatus”.

(IP1)

Several additional thoughts and suggestions pertaining to creating an inclusive and non‐judgmental informative intervention was brought forward, such as avoiding subtle exclusion of non‐coupled persons (single, dating, etc.) and same‐sex couples. PRPs described how their feedback had had direct impact on such perceived heteronormative language: ‘[…] we addressed parts that felt very heteronormative. That came as feedback from us during a meeting and that was changed very quickly and very well. So that's just one example of how the feedback has worked.’ (IP3).

#### 3.3.2 Structure of Texts

Several suggestions for changes in structural components, such as the order or placement of content sections, were brought forward during meetings. PRPs provided suggestions on how to better structure sections on relationships, where it would be made clearer which information is relevant for individuals who are in partnered relationships, and those who are currently dating or having thoughts about future partner relationships. Additionally, length and level of detail provided in materials were discussed at several meetings. While some PRPs expressed a wish to receive as much information as possible, others expressed a risk for future participants being overwhelmed by the scope of material included. A wish for information to be presented in a more manageable way was highlighted and following discussions within the group, content was organised under several expandable headings. To avoid the risk of future participants missing out on relevant information because of this structure, the content was reviewed to ensure that crucial information was placed in the beginning of a new section, and the expandable texts were given clear, descriptive headings.

#### 3.3.3 Intervention Design Elements

Thoughts and suggestions about several design elements were also proposed. PRPs wished for a more dynamic program, with suggestions for various discussion forum features and components to engage and activate future participants. From discussions within the shared working group, a suggestion emerged to produce brief audio‐recorded introductions (so‐called teasers) as a way of introducing and creating interest for each new module. For the upcoming meeting with the shared working group, the researchers produced example teasers for PRPs to review. Following revisions and suggestions on topics and structure of the content, a teaser episode was produced for each module in the Fex‐Can 2.0 intervention.

#### 3.3.4 Future Pilot Trial

Several aspects of the future pilot trial were discussed during the shared working group meetings. First, PRPs provided input on and suggestions for the recruitment of future study participants, such as the language, content and layout of recruitment materials. Following initial input on recruitment material prototypes, the research group revised the material and sent it out to PRPs to be revisited at an upcoming meeting. Additionally, suitable channels for recruitment were suggested, including patient organisations, social media channels relevant for different age groups and rehabilitation centres. Some PRPs further offered to distribute materials within their patient organisations. Content and structure of screening interviews to be used for recruitment of future study participants were discussed, with PRPs emphasising the importance of keeping the information presented during screening interviews brief and focused.

## 4 Discussion

This study provides a detailed account of a long‐term co‐creative process involving young adults diagnosed with cancer in the refinement of an internet‐delivered intervention (Fex‐Can 2.0) targeting sexual problems and fertility‐related distress. Findings underscore the value of sustained, structured, and reflective collaboration with PRPs, highlighting both the potential and the complexity of meaningful PPI, as well as the impact that contributions from PRPs have had on the intervention.

The structured meeting format, including opportunities for social interaction, clear agendas and pre‐distributed materials, as described in the category *Collaborative working process*, was instrumental in fostering a safe and productive environment. This aligns with findings from Nissen et al. [7], who identified well‐structured organisation and explicit goals as critical components of their PPI effort. Nissen et al. [7] further emphasised the importance of open communication about which aspects of a study that can be influenced by PRPs, and what is beyond the scope of the collaboration. Such clarifications may help establish realistic expectations, strengthen trust, and enable PRPs to focus their contributions where they can exert the greatest impact. Similarly, researchers in the present collaboration aimed to continuously inform PRPs about what could and could not be modified, ensuring efforts were directed towards areas within their influence. Notably, previous research has identified insufficient clarity regarding scope of involvement as a barrier to PPI [36].

The present collaboration adopted an iterative approach that enabled continuous feedback and reflection. PRPs valued this approach, and appreciated the opportunity to see how their input was integrated into the intervention. This mirrors findings from our previous PPI work in the development of the original Fex‐Can intervention [28], where explicit acknowledgement and visualisation of contributions were pivotal for enhancing motivation and confidence. Previous research has similarly emphasised that feedback and transparency regarding PRP's input are valued components of successful involvement [37], and may increase motivation for continued engagement [38, 39]. As discussed by Svedin and colleagues [40], insufficient feedback can lead PRPs to feel uncertain about whether their input has had any impact or to remain unclear about their roles in the collaboration.

Efforts to establish a safe group atmosphere, described in category *Group atmosphere*, included the setting of meeting ground rules. Such rules included respect, confidentiality and allowance for emotional expressions, and were determined by group consensus at the first shared working group meeting, in line with NIHR recommendations for collaborative and inclusive working practices [1]. Some PRPs expressed that the durations of meetings were too short. Given that they dedicated parts of their weekends to participate and, in some cases, travelled considerable distances, they requested longer sessions to maximise the value of their involvement. Accordingly, meeting duration was increased, however, it was also important to balance this preference with accessibility, as some PRPs experienced fatigue and pain. This underscores the need for flexibility in PPI work, ensuring that engagement opportunities are meaningful while accommodating participants' needs.

Furthermore, each meeting began with a round of personal check ins, enabling participants to share life updates and PRPs expressed that such practices made them feel welcomed, cared for and respected. This approach reflects findings from previous studies, which underscores the importance of emotional safety and relational connection in PPI [7, 39, 41, 42]. However, close relationships developed through such efforts may also introduce challenges, such as reluctance to voice opposing views or critique decisions due to well‐known power imbalances within PPI [43, 44]. In addition, the researchers' efforts to create a comfortable environment may at times limit the exploration of more challenging topics. To help mitigate such challenges, researchers continuously made explicit that differing perspectives were valued. This is further reflected in the group rules established at the first meeting, which emphasised openness and the value of different perspectives. To support PRPs in expressing their views, future co‐creative collaborations may benefit from offering a range of ways to share perspectives and provide feedback (e.g., written reflections, opportunities for anonymous input). This may help ensure that participants feel comfortable contributing and that a broad range of views is captured. Further, while roles and expectations about the collaboration was discussed in the shared working group at the outset of the collaboration, future co‐creative projects may benefit form revisiting these topics in later sessions to support ongoing reflection on group dynamics and evolving roles.

Previous research has highlighted the positive impact of PRPs on research design (e.g., recruitment, dissemination) [45]. However, few studies explicitly report the impact of PPI on intervention development, underscoring the importance of transparent documentation. The multifaceted input from PRPs in the present collaboration, as described under the category *Concrete impact*, informed changes that may enhance the accessibility, inclusivity and overall relevance of the Fex‐Can 2.0 intervention to its target population. Specifically, PRPs influenced the language and content of the intervention (e.g., improving accessibility), structure of texts (e.g., enhancing readability), intervention design elements (e.g., inclusion of brief teasers) and the future pilot trial (e.g., recruitment materials). Similar impact of collaboration with PRPs were reported in the studies by Nissen et al. [7] and Svedin et al. [40]. Both studies highlighted improvements in intervention content, including enhanced clarity, relevance and comprehensibility of materials, as well as adjustments to layout and structure of texts [7, 40]. Similar to the present collaboration, patient representatives in the study by Nissen et al. [7] further contributed with videos discussing their experiences of cancer late effects to be included in their intervention. Finally, Nissen et al. [7] reported improvements to interview guides and information materials for their future evaluation study. Impact of PPI on recruitment to clinical trials have been reported in previous studies [16], highlighting the importance of involving the target group in the development recruitment materials and strategies. In the present collaboration, PRPs were involved in discussions pertaining to the layout and language of recruitment materials, as well as choice of recruitment channels. Further, some PRPs offered to assist with recruitment by sharing information within their networks. Importantly, in line with previous studies [16], such efforts may hopefully increase enrolment rates.

### 4.1 Methodological Strengths and Limitations

One limitation of the present study is the underrepresentation of certain groups and/or life experiences among the group of PRPs, particularly men, persons of lower education levels and those living in rural areas. A more heterogeneous PRP group would have improved representation and may also have introduced a wider range of perspectives and more opportunities for critical discussion. This reflects broader challenges in achieving diversity in PPI [36, 46]. Recruitment of PRPs for the present collaboration was mainly carried out through patient organisations, as well as via social media to maximise reach, and all interested individuals were screened in an effort to support diversity within the PRP group. However, no strategies specifically aimed at underrepresented groups were implemented. More targeted recruitment approaches, including partnerships with community‐based organisations and culturally tailored outreach, are therefore needed in future collaborations [36, 46, 47, 48]. Further, recruiting PRPs in close collaboration with clinics may enable inclusion of individuals who are more unselected with regard to prior interest, resources, or engagement in research.

Impact logs, field notes, and interviews gave a broad informational base which partly acted as its own data triangulation (several sources expressing the same things), as was the case for previous research studies where multiple data sources allowed for both corroboration of interpretation and enhanced credibility [49, 50]. Further, PRPs were requested to review and provide feedback on the results section to check whether any misinterpretations had occurred, to discuss whether results corresponded with their experiences of the collaboration, and propose alternative labels for categories and subcategories if deemed necessary. A total of four PRPs provided input, and this yielded confirmation that the interpretations were accurate from their perspective, and no new suggestions for categories were brought forward.

To enable PRPs' to freely express their views of the collaboration, the individual interviews were conducted by an independent researcher who had no prior involvement in the research project. Interviewees were also ensured that the research team would not have any access to information about who had been interviewed, or to the raw interview data. Despite this, no negative perceptions of the collaboration were reported by PRPs. Previous studies have shown that PRPs may experience negative impact, such as frustration and feelings of being overburdened [17, 22]. While interviewees in the present study may not have experienced such issues, social desirability bias cannot be ruled out, as participants might have been reluctant to share concerns. It is therefore possible that more critical or negative experiences were unvoiced during interviews. Furthermore, an important limitation is that PRPs who withdrew from the collaboration were not interviewed, which may have resulted in missing more critical perspectives on the collaboration.

Additionally, only four out of the eight PRPs ultimately participated in interviews. While none of the remaining PRPs actively declined, the external researcher responsible for the interviews received no response despite repeated contact attempts. Several factors may have contributed to the lack of response. While the research team members continuously informed PRPs about the interviews and about who would contact them regarding participation, uncertainty about the distinction between the collaboration and the interview process mat have been present. Further, competing demands on participants' time, or simply missed or overlooked communication may have had a role. Although the non‐response limits representation across all PRPs and selection bias cannot be ruled out, field notes and the impact log information include contributions from all PRPs. The consistency between the interview findings and the two other data sources thus supports the overall findings (see Supporting File S2).

### 4.2 Implications for Future Research and Practice

This study seeks to strengthen transparent reporting of PPI processes in health research. By providing detailed accounts of the structure, approaches, and reflections from a long‐term PPI initiative, it addresses the need for richer descriptions of such involvement and its impact. In doing so, it may offer insights into what works, and provide practical considerations for researchers aiming to engage PRPs effectively and meaningfully.

## 5 Conclusions

This study demonstrates the value, feasibility and impact of engaging young adults with a cancer experience as PRPs in revising an internet‐delivered psychoeducational intervention targeting sexual problems and fertility‐related distress. Through structured meetings formats, transparent processes and continuous feedback, the collaboration fostered trust and inclusivity, with PRPs feeling cared for and able to contribute meaningfully to the intervention. Input from PRPs led to concrete improvements aiming to increase the relevance and attractiveness of the Fex‐Can 2.0 intervention, including revisions to language and content, restructuring of texts, intervention design elements, and adaptions to the planned pilot trial.

Findings from the present study contribute to a growing evidence base supporting meaningful PPI in intervention development. Future research should continue to examine the dynamics of long‐term, collaborative partnerships, advance inclusive recruitment approaches for underrepresented groups, and ensure transparent reporting of PPI contributions and their influence on intervention development processes.

## Author Contributions

**Sarah Marklund:** conceptualisation, methodology, formal analysis, investigation, writing – original draft, writing – review and editing, visualisation, project administration. **Rebecca Skog:** conceptualisation, methodology, investigation, writing – review and editing, writing – original draft, project administration, formal analysis. **Lars Sjödin:** conceptualisation, methodology, writing – review and editing, project administration. **Johanna Rose:** writing – review and editing. **Marit Silén:** formal analysis, writing – review and editing. **Lena Wettergren:** conceptualisation, methodology, resources, writing – review and editing, supervision, project administration, funding acquisition. **Claudia Lampic:** conceptualisation, methodology, resources, writing – review and editing, supervision, project administration, funding acquisition.

## Ethics Statement

Regional Ethical Review Board in Stockholm (record no: 2024‐01576‐02, 2025‐02691‐02). Informed consent was obtained from all participants.

## Conflicts of Interest

The authors declare no conflicts of interest.

## Supporting information

Supporting File S1

Supporting File S2

Supporting File S3

Supporting File S4

## Data Availability

The data that support the findings of this study are available from the corresponding author upon reasonable request.
