# Supplementary material for: Navigating Long‐Term Co‐Creative Research With Young Adults Diagnosed With Cancer: A Qualitative Study
Source: Health Expect. 2026 Jun 3;29(3):e70713. doi: 10.1111/hex.70713 (PMC13240287; doi:10.1111/hex.70713)
Supplement: Supplementary file 1 — Supporting File S1 [file HEX-29-e70713-s002.docx]

| **Impact log – Patient and public involvement in the Fex-Can 2.0 project** | | | | | |
| --- | --- | --- | --- | --- | --- |
| **Date, time and place** | **Type of activity and involvement** | **Present/absent** | **Ideas and suggestions** | **Impact** | **Reflection and next steps** |
|  |  |  |  |  |  |
|  |  |  |  |  |  |
|  |  |  |  |  |  |
|  |  |  |  |  |  |
|  |  |  |  |  |  |
|  |  |  |  |  |  |
|  |  |  |  |  |  |
|  |  |  |  |  |  |
|  |  |  |  |  |  |
|  |  |  |  |  |  |
