# Supplementary material for: Navigating Long‐Term Co‐Creative Research With Young Adults Diagnosed With Cancer: A Qualitative Study
Source: Health Expect. 2026 Jun 3;29(3):e70713. doi: 10.1111/hex.70713 (PMC13240287; doi:10.1111/hex.70713)
Supplement: Supplementary file 2 — Supporting File S2 [file HEX-29-e70713-s001.docx]

| **Supplementary table S2.** Excerpts from field notes, impact logs and interviews | | | |
| --- | --- | --- | --- |
| **Category: Collaborative working process** | | | |
| **Subcategory** | **Field notes** | **Impact log information** | **Interviews** |
| *Forms of collaboration* | Feedback regarding the meeting:  Great, a culture of open dialogue and flexibility, breaks let one feel refreshed throughout, exciting to see how it will be next time, good food. (Meeting 1). | A good day, important to work through what we discussed today. Good that we did not spend too much time going through information as we have done at earlier meetings. It has also worked well for those who have not attended all meetings. (Meeting 4). | “Well, it has been very straight forward. You get a home assignment before you get there. You talk about the assignment and discuss some feedback. And the you work, step by step, through the program. That is what we have done during the meetings… So, it has, I think, worked really well because it has been very focused.” (IP3). |
| *Feedback and transparency* | Discussion about last meeting:  We mentioned that we will provide feedback on the changes that you have suggested, and also go through the changes that we have made based on your input and comment, to anchor it with you and ensure that we have interpreted everything correctly. (Meeting 2). | XX talks about the three first meetings that we had and what we have done so far, as well as about what we are doing right now and will be doing in the near future. Among other things, this includes the work to incorporate the input from PRPs into the intervention. (Meeting 4). | “They’ve started pretty much every meeting with, okay, “the last, or the two second last times, we have focused on this and that” and they have summarized and confirmed with us “did we understand you correctly?”. [I’ve] strongly appreciated that they confirmed with us.” (IP4). |
| *Efficient ways of working* | it’s hard to read when you are sitting together, better to read before…Send out all texts beforehand that we are meant to work through during the meetings (“these are texts that we will read through at the meetings”) so those that want to can read at home. (Meeting 2). | Just enough material for the home assignment, sometimes it has been quite a lot…Easier to have deeper discussions when there are less materials. (Meeting 6). | “Since time is limited when we meet it is important that we have prepared too. Listen to some link, read some materials, provide feedback. It you haven’t been able to attend you could still provide some feedback on the material. So, I thought that worked very well with how it was organized.” (IP4). |
| **Category: Group atmosphere** | | | |
| **Subcategory** | **Field notes** | **Impact log information** | **Interviews** |
| *Diverse working group* | [PRP name] says that “there is always too much focus on people who are in a relationship – so much is focused on them all the time. I will fight for more for focus on those who are single.  (Meeting 3). | Add more social support, other types of relationships, rather than only romantic relationships. (Meeting 3) | “It felt like they approached us in a very good way. We are all very different and extremely different people with a common denominator. I don’t know, several times I have thought that I would like to meet them and go out for dinner with them sometime.” (IP2). |
| *Supportive working environment* | It’s very good to come here and talk with others and exchange experiences. (Meeting 6). | The meetings are a bit therapeutic, it’s good because it makes you reflect and share with others. (Meeting 4). | “We PRPs have really shared some very private, deep, emotional, hard, fun [things]. Everything really, high and low. So, it has like almost felt like a little support group. A bit therapeutic in some instances.” (IP2) |
| **Category: Concrete impact** | | | |
| **Subcategory** | **Field notes** | **Impact log information** | **Interviews** |
| *Language and content* | PRPs describe getting stuck on the word “reproduction apparatus (p.2) – it’s not bad, but weird. We ask what they would like instead. PRPs suggest: “reproductive organs, anatomy.” (Meeting 2). | Got stuck on “reproduction apparatus”, suggest “reproductive organ” or “anatomy” instead (Meeting 2). | “There, I really feel that we have had great impact. We have, I mean during the meetings, we have specifically said that there were some parts that felt very heteronormative. So, it came up during our meeting and it was changed really quickly and really good.” (IP3). |
| *Structure of texts* | Suggestion for more texts where you can “click” to read it, rather than being “fed” it right away. For example, links to “More about orgasm”, “more about anatomy” and so on...The texts in the beginning should be shorter and more general. (Meeting 2) | PRPs suggests that we include links under each text to divide the text better and further guide the participant through the materials, have clickable links, digital signposts. (Meeting 2). | “Yes, but a lot about choice of words and how the texts are structured…It really comes down to it [the intervention] utilizing good texts, that it is inviting, and that it is undemanding to the extent it can. If it is only heavy, long texts, then you easily lose your motivation.” (IP1). |
| *Intervention design elements* | What would have made you stay in the program? Images, opportunity to listen to things, variation (it should happen a lot of things, not just texts, but rather great variation of the content). (Meeting 3) | PRPs suggest short videos within the anatomy texts, even though the pictures already used are considered evident. (Meeting 1). | “I really think that working on these things together will benefit the patients and surely increase motivation to continue working with the program and read everything. I think it is an incredibly good initiative and important.” (IP1). |
| *Future pilot trial* | PRPs were generally very positive towards the recruitment materials, appreciated the images and layout. Regarding language/texts they suggested that we avoid the word *problems*, better to use *challenges*, avoid value-laden words. (Meeting 6). | Include a checklist in the ad, describing important traits of the participant – ”if you are x, y, z: register here”. The ad for recruiting us [the PRPs] was well done, make something similar. Highlight that you get help at the same time as helping research. (Meeting 4). | “And the material has been great, the information materials. Very easy to take to heart and understand. And the intent. Very good information.” (IP1). |
