# Supplementary material for: Navigating Long‐Term Co‐Creative Research With Young Adults Diagnosed With Cancer: A Qualitative Study
Source: Health Expect. 2026 Jun 3;29(3):e70713. doi: 10.1111/hex.70713 (PMC13240287; doi:10.1111/hex.70713)
Supplement: Supplementary file 3 — Supporting File S3 [file HEX-29-e70713-s004.docx]

| **Guidance for Reporting Involvement of Patient and the Public** (GRIPP2-SF) | | |
| --- | --- | --- |
| **Section and topic** | **Item** | **Page No** |
| 1: Aim | Report the study aim | 4 |
| 2: Methods | Provide clear description of the PPI in the study | 4, 9-11 |
| 3: Study results | Outcomes – Report the results on PPI in the study, including both positive and negative outcomes | 12-14 |
| 4: Discussion and conclusion | Outcomes – Comment on the extent to which PPI influenced the study overall. Describe positive and negative effects | 14-16 |
| 5: Reflections and critical perspective | Comment critically on the study, reflecting on the things that went well and those that did not, so others can learn from this experience | 16-17 |
| PPI=patient and public involvement | | |
